# HIV Tat as a latency reversing agent: turning the tables on viral persistence

**DOI:** 10.3389/fimmu.2025.1571151

**Published:** 2025-04-11

**Authors:** Bridget M. Fisher, Paula M. Cevaal, Michael Roche, Sharon R. Lewin

**Affiliations:** ^1^ Department of Infectious Diseases, The University of Melbourne at The Peter Doherty Institute for Infection and Immunity, Melbourne, VIC, Australia; ^2^ ATRACT Research Centre, Infectious and Inflammatory Diseases Theme, School of Health and Biomedical Sciences, RMIT University, Melbourne, VIC, Australia; ^3^ Victorian Infectious Diseases Service, The Royal Melbourne Hospital at the Peter Doherty Institute for Infection and Immunity, Melbourne, VIC, Australia; ^4^ Department of Infectious Diseases, Alfred Hospital and Monash University, Melbourne, VIC, Australia

**Keywords:** HIV, latency, Tat, latency reversal agent, HIV cure

## Abstract

The ‘shock and kill’ approach to an HIV cure involves the use of latency reversing agents (LRAs) to reactivate latent HIV, with the aim to induce death of infected cells through virus induced cytolysis or immune mediated clearance. Most LRAs tested to date have been unable to overcome the blocks to transcription elongation and splicing that persist in resting CD4+ T cells. Furthermore, most LRAs target host factors and therefore have associated toxicities. Therefore, there remains a high need for HIV-specific LRAs that can also potently upregulate expression of multiply-spliced HIV RNA and viral protein. The HIV Transactivator of Transcription (Tat) protein plays an important role in viral replication - amplifying transcription from the viral promoter - but it is present at low to negligible levels in latently infected cells. As such, it has been hypothesized that providing Tat in *trans* could result in efficient HIV reactivation from latency. Recent studies exploring different types of Tat-based LRAs have used different nanoparticles for Tat delivery and describe potent, HIV-specific induction of multiply-spliced HIV RNA and protein *ex vivo.* However, there are several potential challenges to using Tat as a therapeutic, including the ability of Tat to cause systemic toxicities *in vivo*, limited delivery of Tat to the HIV reservoir due to poor uptake of nucleic acid by resting cells, and challenges in activating truly transcriptionally silent viruses. Identifying ways to mitigate these challenges will be critical to developing effective Tat-based LRA approaches towards an HIV cure.

## Introduction

Following cessation of antiretroviral therapy (ART) in people with HIV (PWH), HIV rapidly rebounds from a pool of latently infected cells (1, 2). Latently infected cells contain an integrated intact provirus but express minimal HIV RNA and proteins, resulting in a long-lived persistent reservoir (3). Strategies towards an HIV cure have focused on reducing the number of latently infected cells such that PWH can maintain undetectable viral loads in the absence of ART [reviewed in (4)]. The ‘shock and kill’ approach involves the use of latency reversing agents (LRAs) to upregulate HIV RNA transcription and protein expression, resulting in the death of an infected cell by immune-mediated clearance or virus-mediated cytotoxicity [reviewed in (5)].

Several classes of LRAs with distinct mechanisms of action have been described, and many have demonstrated strong induction of HIV RNA *in vitro* and *ex vivo* (6–10). Clinical trials have since been conducted to assess the performance of these *in vivo*, of which a common primary endpoint for efficacy has been cell-associated, unspliced HIV RNA as a measure of HIV reactivation (see (11) for a systematic review). Although some LRAs both alone and in combination were able to increase unspliced HIV RNA in PWH on ART, few demonstrate a decrease in HIV DNA and/or the replication competent inducible HIV reservoir, indicating a need for more potent LRAs with novel mechanisms of action (11–14).

This lack of potency of current LRAs is thought to be due to two reasons. Firstly, whilst most current LRAs can upregulate transcription initiation, few if any, are able to overcome the subsequent blocks to transcription elongation, completion and splicing (15–17). As splicing is a pre-requisite for HIV protein production, it is considered the best predictor of efficient latency reversal (18), meaning any LRA must be able to potently upregulate multiply-spliced HIV RNA. Secondly, multiple LRAs have been shown to cause various adverse effects as they target host pathways and therefore have effects on cellular transcription and often undesirable effects on immune responses (14, 19, 20). Since PWH can live long, healthy lives on ART, any potential HIV cure approach must minimize toxicities while maximizing efficacy (21, 22). In this review, we focus on the rationale, feasibility and findings of utilizing the HIV Transactivator of Transcription (Tat) protein as a next-generation LRA and discuss strategies for maximizing potency and minimizing toxicities.

## Tat structure and function

Tat is a small, basic protein of about 14-16 kDa, which is encoded by two exons that are alternatively spliced to become a full-length protein (23). Tat most commonly spans 101 amino acid residues (Tat_101_) (24), but a premature stop codon at residue 87 frequently occurs, encoding a truncated Tat_86_ variant (25). The functional domains of Tat largely reside in the first coding exon (amino acids 1-72; Tat_72_) such that it can function independently of the second coding exon (26, 27). The proline-rich domain, or N-terminus, mediates Long Terminal Repeat (LTR) transactivation through interactions with positive transcription elongation factor b and is mostly hypervariable except for a highly conserved tryptophan at position 11 (Trp11). In contrast, the cysteine-rich domain contains a highly conserved string of cysteines at positions 22, 25, 27, 30, 31, 34 and 37 and mutations at these sites can significantly affect Tat function (28). The third domain spans residues 38-48 and contains a hydrophobic core sequence. Together, the first three domains compromise the minimal transactivation region which itself is sufficient for transactivation capability (29). The remainder of the first coding exon, referred to as the basic region, is comprised of the Transactivation Response Element (TAR)-binding motif (_49_RKKRRQRRR_57_) (30) and a glutamine rich region which is implicated in Tat-mediated apoptosis of T cells (31). Amino acids 31-61 within the first coding exon have also been shown to be related to Tat-related morbidities (32). Although the transactivation domain is localized to Tat_72_, the second coding exon plays a role in the control of HIV transcription in CD4+ T cells by kappa-light-chain enhancer of activated B cells (NF-κB) (33). The second coding exon largely contributes to viral infectivity and is essential for efficient replication of macrophage-tropic HIV strains (34). This region also contains a _73_RGD_75_ motif which allows for interactions with cell surface molecules, triggering intracellular signaling cascades (35).

**Figure 1:**
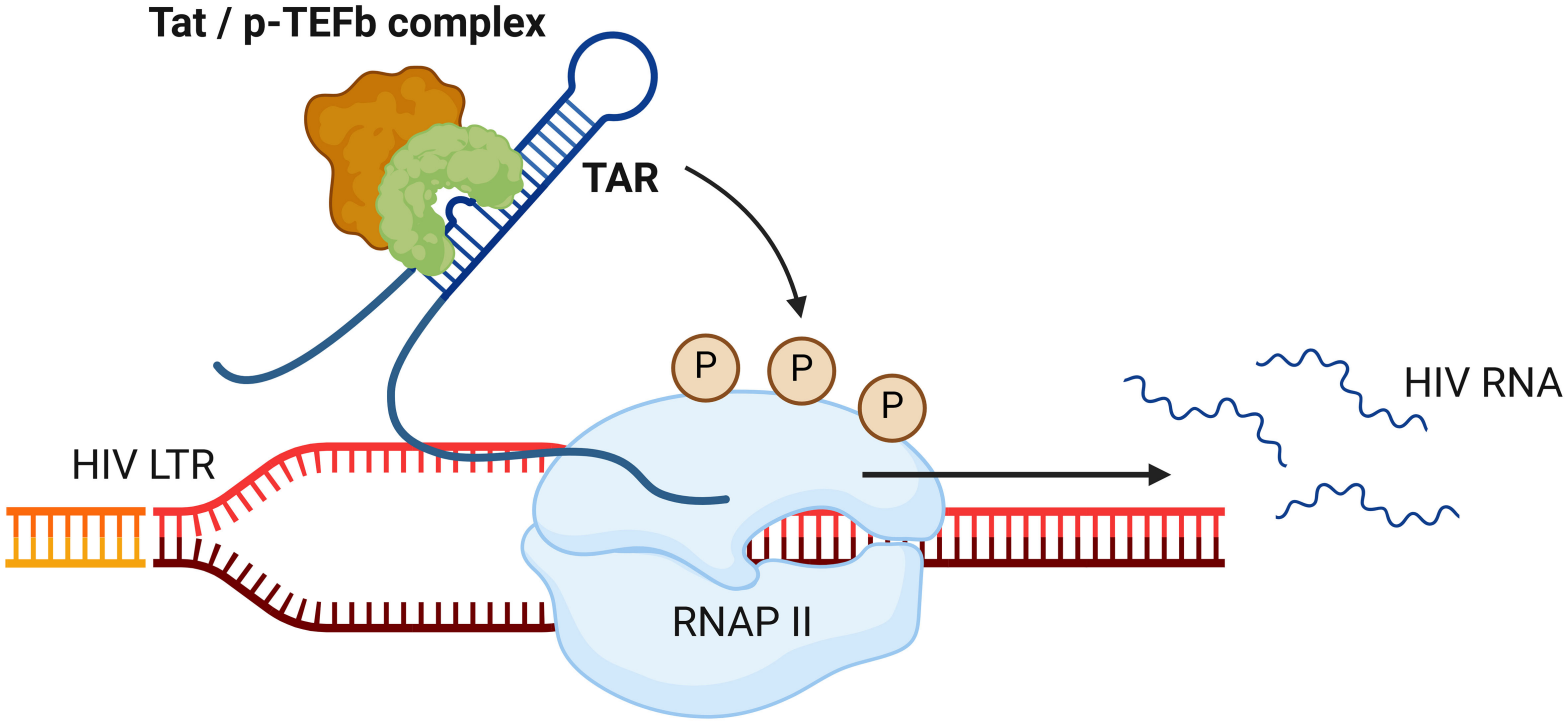
Tat-mediated enhancement of RNA polymerase II processivity. Following its translation, the HIV transactivator of transcription (Tat) protein forms a complex with positive transcription elongation factor b (p-TEFb) and binds to the transactivating-response (TAR) element within the early HIV transcript. This causes the hyperphosphorylation (P) of RNA polymerase II (RNAP II), enhancing the transcription processivity of the enzyme. This process is essential for the production of full-length unspliced HIV RNA; and multiply-spliced HIV RNA and virion production thereafter.

HIV transcription is driven by the Tat protein, which amplifies expression from the viral promoter within the HIV LTR (36). By binding to the TAR hairpin in the nascent RNA strand, Tat recruits p-TEFb (37), resulting in the hyperphosphorylation of RNA polymerase II and increased transcriptional processivity from the LTR (**Figure 1**) (38). This is essential for the production of full-length unspliced HIV RNA, which can be spliced into singly spliced and multiply-spliced HIV RNA which are translated into the various HIV proteins for virion formation. In latently infected cells, Tat levels are reduced, rendering viral transcription low or silent, whilst in productively infected cells, Tat levels are elevated and therefore enhance HIV transcription (39, 40). Even when a cell is in a resting state, stochastic fluctuations in Tat expression can drive a cell to alternate between productive infection or latency (40, 41), which is sufficient to overcome cell-driven silencing of HIV transcription (42).

### Mechanisms of Tat secretion and uptake

**Figure 2:**
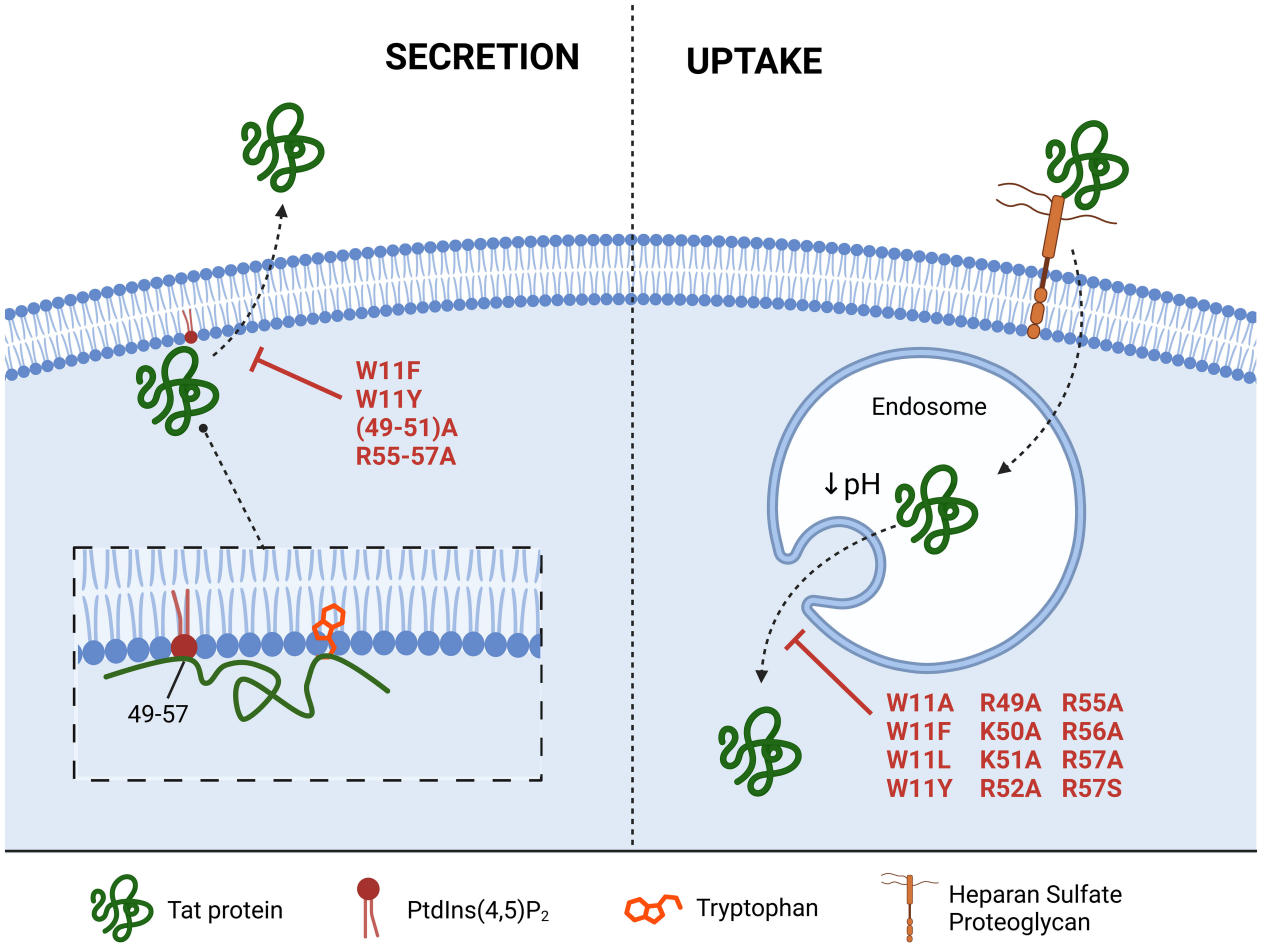
Impact of mutations on Tat secretion and uptake. Tat secretion occurs through interactions between phosphatidylinositol-4,5-bisphosphate (PtdIns(4,5)P_2_) the Tat basic domain (residues 49-57), and a conserved tryptophan at position 11 (Trp11). Uptake occurs through the binding of Tat to heparan sulfate proteoglycans, followed by uptake into endosomes. Point and combinatorial substitution mutations in both the basic domain and Trp11 reduce secretion and uptake efficiency of Tat, albeit to different degrees.

Tat secretion occurs via an unconventional secretion pathway (43), which is initiated by binding of the protein to phosphatidylinositol-4,5-bisphosphate (PtdIns(4,5)P_2_), a phospholipid component of the inner leaflet of the plasma membrane (**Figure 2**) (44). These interactions are mediated by the basic domain of Tat and the conserved Trp11 within the first coding exon. Trp11 inserts into the plasma membrane as a pre-requisite for secretion (45).

Tat also contains a protein transduction domain which allows it to penetrate cells from the extracellular environment (46). This mechanism of entry is so efficient that multiple groups have used this cell penetrating peptide sequence to deliver a large variety of cargo to a cell, unrelated to HIV (47–49). Indeed, purified Tat protein is able to transactivate the HIV LTR when added to the extracellular environment, highlighting its ability to cross both the plasma and nuclear membranes (50, 51). This internalisation is dependent on Tat binding to heparan sulfate proteoglycans (HSPGs) on the cell surface, resulting in uptake into endosomes that gradually acidify, leading to protein release (52). The conserved Trp11 residue also plays an essential role in Tat release from the endosome during the engulfment of extracellular Tat (53).

### Tat secretion and systemic distribution *in vivo*

Tat can be secreted from cells, potentially resulting in its dissemination throughout the circulation to multiple cell types and tissues (43, 44, 54). Indeed, secreted Tat has been detected in the cerebrospinal fluid (55, 56) and sera (57–61) of PWH, even in individuals on effective ART with undetectable viral loads.

Tat expression in the central nervous system (CNS) could be from HIV DNA+ cells in the brain, or *via* the trafficking of Tat protein from the periphery across the blood brain barrier (62). In mice where modifications resulted in Tat being secreted from β cells of the pancreas, Tat was able to distribute widely to the brain, thymus, spleen, heart, lung, kidney, liver and pancreas as well as resting CD4+ T cells (63). Therefore, systemic effects of Tat need to be considered and minimised when using Tat as an LRA.

### Tat-related toxicities

Many studies have shown differential expression of genes and proteins in cells after Tat treatment [reviewed in (64)]. Tat is known to modulate the expression of multiple cellular genes including interleukin (IL)-6, tumour necrosis factor (TNF) β, interferon regulatory factor (TRF) 7, IL-2 and cluster of differentiation (CD) 69 by binding to TAR-like sequences and promoter regions or interacting with host transcription factors (65–69). Other studies have shown that expression of HIV Tat can upregulate the expression of human endogenous retroviruses (70–72).

Tat concentrations as low as 10-15 ng/mL in the sera produce significant biological effects such as DNA damage in B cells, contributing to an increased risk of Burkitt’s lymphoma in PWH (57). The presence of Tat can induce dysfunction in microglia, CD4+ T cells, astrocytes, neurons and cardiomyocytes following cellular uptake (73–76). Tat can also act as a chemokine, attracting monocytes and macrophages into areas of productive HIV infection, resulting in localized inflammation (77). The chronic persistence of Tat in the CNS is important as Tat can contribute to the development of HIV-associated neurocognitive disorder [reviewed in (78, 79)].

## Tat as a potent, HIV-specific latency reversing agent

Initial studies using transfection of a Tat expression plasmid resulted in weak viral reactivation in a latently infected cell line (80). It was later shown that cells cultured in the presence of purified recombinant Tat protein were unable to establish latency, suggesting that exogenous Tat can enter the nucleus from the extracellular environment to help drive productive infection (50). Since Tat’s activity relies on the expression of the HIV TAR element (37), this approach would likely have a higher degree of HIV-specificity compared to other LRAs which typically target host transcriptional pathways. Given Tat’s important role in overcoming blocks to transcription elongation, it has been hypothesized that providing Tat in *trans* could result in efficient HIV reactivation, and thus Tat may represent a highly potent and specific latency reversing agent (42).

### Recombinant Tat protein

Recent work demonstrated that a truncated Tat variant comprising 66 amino acids (T66) was found to have comparable transactivation activity to both Tat_72_ and Tat_86_ in HEK293T cells expressing LTR-driven reporter plasmids (51). T66 also induced HIV RNA expression in an *in vitro* model of HIV infection and in CD4+ T cells from PWH on ART, similar to positive controls that activated the T-cell, including Phorbol myristate acetate (PMA), phytohemagglutinin (PHA) and anti-CD3/anti-CD28 stimulation (51). Furthermore, T66 protein in CD4+ T cells from PWH on ART did not significantly induce global cellular activation as seen with PHA (51). Only eight genes were identified as being differentially expressed following T66 treatment compared to non-stimulated controls, indicating minimal perturbation to the host cell transcriptome (51).

The ability of T66 protein to work equal to - or better than - the current gold-standards for latency reversal *in vitro* with few off-target effects highlights its promise as an LRA candidate. However, administering exogenous purified Tat protein in humans is likely problematic due to limited *in vivo* stability and the concerns of toxicities relating to systemic Tat protein (81). The successful application of Tat as a next-generation LRA will require an efficient delivery mechanism.

### Exosomes containing Tat protein

The field of nanotechnology may play an important role in addressing challenges related to efficient Tat delivery *ex vivo* and *in vivo*. The first approach describing nanoparticles for the delivery of Tat as an LRA utilized Tat_101_ protein encapsulated into exosomes (EXO-Tat) (82). Measuring 30 to 150 nm in diameter, exosomes are the smallest type of extracellular vesicle and can be used for various therapeutic purposes due to their ability to encapsulate complex protein, lipid and nucleic acid cargoes (83). EXO-Tat increased unspliced HIV RNA *ex vivo* in CD4+ T cells from PWH on ART, but at a lower level than T-cell activation using PMA/ionomycin (82). EXO-Tat also upregulated multiply-spliced HIV RNA in 80% of donors, with potency exceeding that of current-generation LRAs panabinostat (30 nM) and disulfiram (500 nM) (82). Treatment with EXO-Tat in combination with either one of these LRAs further increased HIV RNA by 30-fold compared to EXO-Tat alone (82). HIV protein production was also observed in 50% of donors tested, even though all donors expressed p24 following PMA/ionomycin stimulation (82). However, treatment with EXO-Tat resulted in significant perturbations in the host cell proteome, upregulating over 30% of identified cellular proteins associated with translational machinery and metabolic pathways (84). EXO-Tat was also linked to upregulation of proteins associated with oxidative stress and apoptosis (84). These findings agree with multiple other studies showing differential expression of genes and proteins in various cell lines after Tat treatment (85–88). This calls into question the suitability of utilizing EXO-Tat as an LRA.

### Lipid nanoparticles encapsulating mRNA encoding Tat

More recently, lipid nanoparticles (LNPs) encapsulating messenger mRNA (mRNA) encoding Tat have been developed. LNPs encapsulating mRNA encoding the T66 protein (T66-LNPs) could upregulate multiply-spliced HIV RNA and protein production in latently infected CD4+ T cells *ex vivo* (median 188- and 185-fold increase compared to untreated, respectively), with potency similar to that of PMA stimulation (51). T66-LNPs also induced p24 expression, similar to PMA treatment (51). T66-LNPs synergized with classical LRAs including the second mitochondrial-derived activator of caspases (SMAC) mimetic AZD5582 and the histone deacetylase inhibitors panabinostat and vorinostat, leading to a significant increased median fold change in TAR, elongated LTR, polyadenylated and tat-rev transcripts compared to each drug alone (89, 90). Synergistic effects on p24 production were also observed (90). Such findings are important given that it is probable that not all proviruses will be responsive to Tat alone *in vivo* due to the various cellular factors that maintain latency. However, the continued use of classical LRAs negates the benefits of using an HIV-specific LRA.

## Considerations and mitigations strategies pertaining to Tat as an LRA

**Figure 3:**
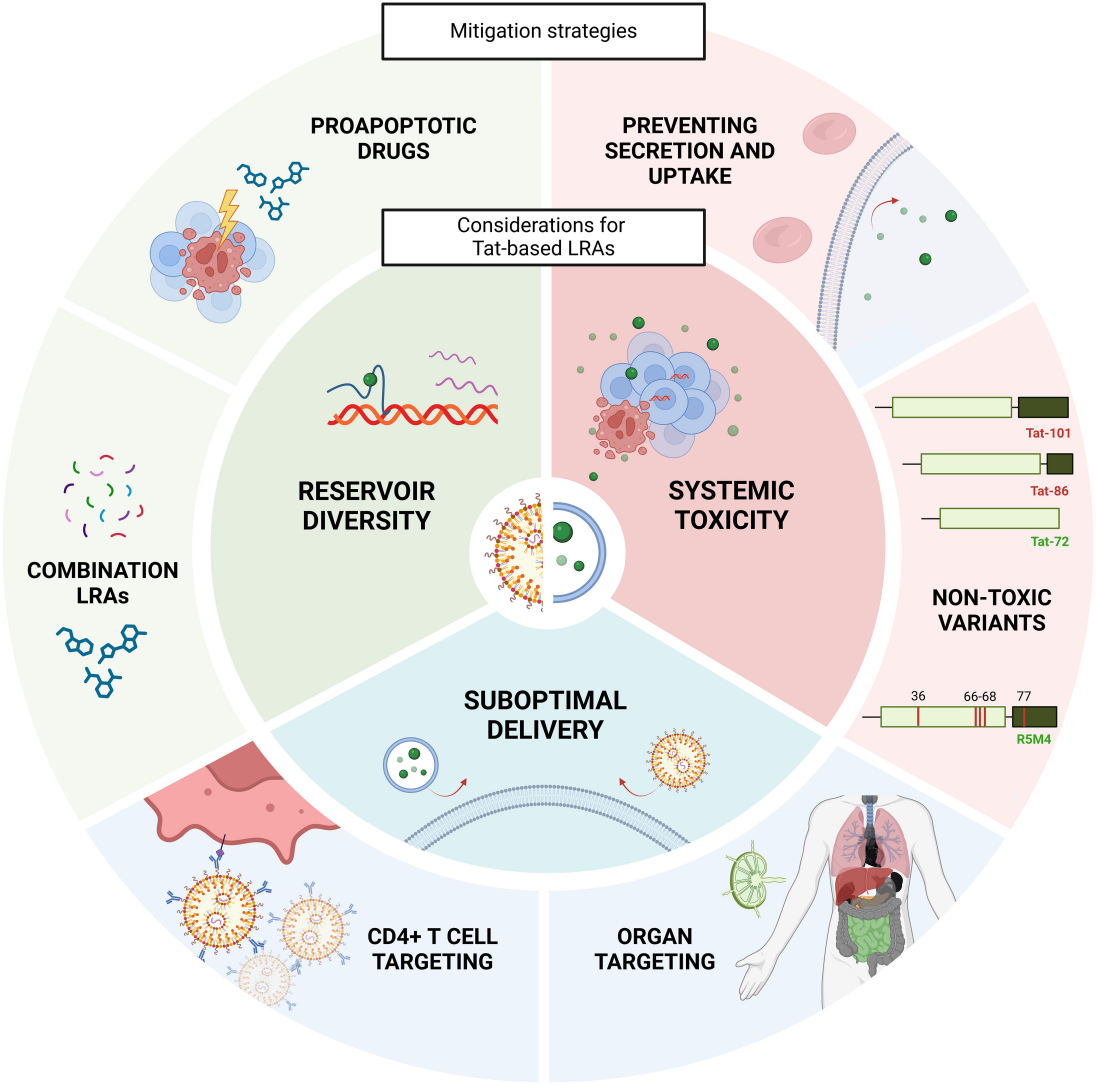
Considerations for the development of Tat-based LRAs. There are multiple potential challenges to the development of Tat-based therapeutics, including systemic toxicities *in vivo*, suboptimal delivery of Tat to latently infected cells, and reservoir diversity, rendering a proportion of proviruses unresponsive to Tat treatment (inner circle). These could be mitigated in several ways (outer circle) including preventing Tat secretion and uptake, utilizing a non-toxic Tat variant, targeting Tat delivery vehicles to CD4+ T cells and anatomical sites of the HIV reservoir; and combining Tat with other HIV-specific latency reversing agents (LRAs) or pro-apoptotic drugs to drive selective killing of HIV DNA+ cells.

These studies demonstrate promise for the use of Tat as an LRA, given Tat’s ability to potently increase HIV transcription, upregulate expression of multiply-spliced HIV RNA, and act through an HIV-specific mechanism. However, there remain significant challenges to the use of Tat as an LRA (summarized in **Figure 3**) relating to the toxicity of Tat, effective delivery of Tat to the latent reservoir, and reservoir diversity impacting Tat-mediated latency reversal and cell death.

### Systemic toxicities may occur after Tat administration

Targeting Tat to CD4+ T cells could potentially minimize Tat-related toxicities, by reducing Tat expression in cells not infected with HIV and reducing circulating systemic levels of Tat. However, direct delivery to T cells may not mitigate the secretion of Tat. For example, using exosomes to deliver Tat protein may inadvertently enhance Tat secretion. Using a cell culture method of producing EXO-Tat, Tat-expressing HEK293T cells released exosomes containing Tat which were then captured for downstream experiments (82). To effectively produce EXO-Tat via this method, the protein needed to be modified to ensure that once expressed, it was targeted towards the intracellular membrane compartment (82). The result was a Tat mutant that favored its own secretion into exosomes, which could then disseminate *in vivo*. Secretion of unmodified Tat protein could also occur after Tat-LNP treatment, and therefore strategies will be needed to minimize secretion through site directed mutation.

Various mutational studies have identified specific residues of the protein that can prevent Tat secretion and mitigate off-target effects (**Figure 2**). For example, replacing RKK at positions 49-51 with alanine reduced secretion of Tat to 1% of wildtype (44), but in turn impacted transactivation due to the essential role of these basic residues in TAR-binding (45). Similar mutations on the C-terminal end of the basic domain (55-57A) reduced secretion to 30% of wild type (45). Altering Trp11 also impacted the stability of interactions between Tat and PtdIns(4,5)P_2_ (91). For example, replacing Trp11 with phenylalanine or tyrosine prevented Tat secretion from Jurkat cells by 80% compared to wildtype (44).

Point mutations within the Tat basic region and Trp11 have also been employed to prevent Tat uptake by bystander cells. Tat mutations at W11A, W11F, W11L and W11Y decreased transactivation capacity when added to the extracellular environment of Jurkat cells, indicating a reduced ability to reach the cytoplasm (53). Furthermore, changing even a single lysine or arginine to alanine within the basic domain reduced Tat uptake as a cell penetrating peptide (92). Tat sequence and function can also vary among HIV subtypes, including effects on uptake. For example, Tat subtype C has a naturally occurring polymorphism, R57S, in its basic domain, which led to a 70% reduction in uptake compared to Tat subtype B (45).

The length of Tat also has varying effects on toxicity. Tat_72_ lacks the _73_RGD_75_ motif that permits transmigration across the blood brain barrier (93), potentially limiting its dissemination to the CNS *in vivo*. To reduce Tat-related cytotoxicity and immunogenicity, it is also possible to alter the protein domains associated with such toxicities. Firstly, the R57S substitution reduced the induction of proinflammatory cytokine genes TNFα, IL-6, IL-8, IL-1β and chemokine (C-X-C) motif ligand 1 (CXCL1) in response to reduced efficiency of uptake (45). Secondly, an engineered form of Tat_86_, R5M4, with point mutations V36A, Q66A, V67A, S68A and S77A reduced both total cell toxicity and the ability to induce inflammatory cytokine production while having no effect on transactivation potency (94). When injected intravenously into wildtype BALB/c mice at 40 mg/kg, Tat-R5M4 caused no change in liver and kidney function (94). It may therefore be advantageous to incorporate these mutations when using Tat as an LRA.

In summary, there are many residues that have been identified that could be mutated to prevent the secretion and uptake of Tat and its ensuing toxicities. However, it will be key to ensure that any mutations made will not impact the transactivation capacity of the protein, ensuring potency is maintained whilst minimizing toxicities.

### Suboptimal delivery of Tat to the latent reservoir

A common limitation in exosome and LNP delivery systems is that they are unable to efficiently deliver cargo to resting CD4+ T cells. For example, EXO-Tat could only successfully transfect 13% of resting CD4+ T cells in an *in vitro* model of infection (82). Therefore, additional modifications will likely be required to deliver nanoparticles to latently infected CD4+ T cells in circulation and tissue.

The major tissue distribution of systemically administered exosomes in mice include the liver, spleen, kidney, lung and gastrointestinal tract, all of which can be altered by various factors such as the cellular origin of the exosomes, the exosomal membrane composition (*eg*. protein, lipids and glycan), and the pathophysiological conditions of the host (95–101). Furthermore, once exosomes are administered, they are rapidly engulfed by circulating phagocytic cells, which could further impede efficient delivery of Tat to the HIV reservoir *in vivo* (102).

LNPs can also rapidly accumulate in the liver after administration, thereby reducing their potency *in vivo* (103). Studies of T66-LNPs have not reported details of the lipid components of the proprietary LNP nor the resulting transfection efficiency in CD4+ T cells (51, 89, 90). However, others have demonstrated poor transfection efficiencies of CD4+ T cell with non-targeted LNP formulations (104–106). Indeed, to ensure successful transfection, activation of the T cells appears to be a pre-requisite (107). As T cell activation should be avoided as an LRA strategy, some form of targeting is likely to be required to ensure effective protein delivery to resting CD4+ T cells.

#### Targeting Tat to latently infected CD4+ T cells

The targeting of nanomaterials towards particular cells can be achieved *via* two routes: passive targeting or active targeting. The first approach relies on the physicochemical properties of the LNP, including lipid composition as well as the preparation method, size and surface charge which can alter *in vivo* biodistribution (108–110). Active targeting instead involves the addition of specific ligands or antibodies to the nanoparticle surface, which can bind to receptors expressed by target tissues or cells, ensuring precise delivery (111–113).

The ability of CD4+ T cells to undergo receptor-mediated endocytosis, even in a resting state, highlights a potential role for active targeting to ensure efficient Tat uptake. To improve protein delivery of EXO-Tat to resting CD4+ T cells, the C terminus of IL-16, the natural ligand for the CD4 receptor, was conjugated to the extracellular domain of the exosomal protein Lamp2b (82). This approach improved Tat protein expression within resting CD4+ T cells *ex vivo* by 20-fold compared to unmodified EXO-Tat, and induced p24 production in all donors compared to only 50% of donors when using unmodified EXO-Tat, suggesting a previous block to efficient reactivation was due to inefficient Tat protein delivery (82).

Similar targeting approaches have also been utilized by groups employing LNPs for delivery of mRNA to CD4+ T cells. CD4-targeted LNPs, using CD4 antibodies, resulted in a 30-fold higher signal of the reporter mRNA in CD4+ T cells isolated from the spleen in mice, compared to non-targeted LNPs (106). Intravenous injection of the CD4-targeted LNPs into mice resulted in mRNA delivery to 60% and 40% of CD4+ T cells in the spleen and lymph nodes, respectively (106). However, as CD4 is a surface receptor that is not internalised, it is possible that other receptors expressed on the cell surface, such as CD7, transferrin receptor, CD90 and IL-2R may make better candidates for LNP internalisation in T cells (114). CD3 has also been targeted previously to efficiently deliver reporter mRNA to T cells *in vitro* and in mice, however, this was associated with complex immunological consequences and therefore not suitable for therapeutic application in humans (104).

#### Targeting Tat to anatomical sites of the latent reservoir

The other approach to optimize Tat as an LRA is to enhance delivery to key tissues, where the majority of latently infected cells reside in PWH on ART, such as lymph nodes or the gastrointestinal tract (115). LNP targeting to peripheral lymph nodes has been extensively explored in different contexts related to vaccination and therapeutic delivery. For example, to optimize uptake of a vaccine in lymph nodes, the chemical structure of the lipids, charge and size have been altered (116), to allow for the most efficient trafficking after subcutaneous injection (117). This route of administration is less relevant for HIV cure, which relies on widespread dissemination of treatment to all lymph nodes. Instead, conjugation of an antibody that binds to a high endothelial venule marker (MECA-79) which recognizes peripheral lymph nodes addressin on the high endothelial venules of lymph nodes, led to active targeting of microparticles to the lymph node after intravenous administration (118). This strategy could potentially be adapted for the delivery of LNPs to the HIV reservoir.

Targeting the gut-associated lymphoid tissue (GALT) with nanoparticles has also been achieved in a variety of ways, including through the use of lipid-polymer nanoparticle hybrids to enhance the association to the Peyer’s patches (119). Unlike in lymph node targeting strategies, most approaches for enhancing GALT delivery rely on oral administration, which is complicated by physiological barriers of the digestive system resulting in nanoparticle degradation or excretion (120).

LNP transport to the CNS has been shown to be even more difficult due to the high-resistance tight junctions within the brain capillaries that restrict brain uptake of small molecules from the periphery (121). Although exosomes have been shown to cross the blood brain barrier from the circulation (122), additional modifications were needed to deliver LNPs to the CNS. These have included intracerebroventricular or intracerebral injection of the LNPs (123, 124) or the use of antibody-conjugated LNPs to ensure that LNPs traverse the endothelial cells lining blood vessels in the brain, rather than simply transfecting the endothelial cells themselves (125). Alternatively, the lack of CNS penetration by LNPs may be beneficial to a Tat-based LRA, avoiding Tat expression and viral reactivation in the brain whilst achieving both in the periphery.

Although Tat is known to be a highly specific and potent reactivator, further targeting it to only the cells or tissues in which it needs to be expressed will further improve HIV-specificity and potency. However, given the large number of requirements for both T cell and organ targeting, it is unlikely that one LNP formulation alone will be able to simultaneously target mRNA encoding Tat to all of the required sites *in vivo*. Rather, a single formulation which combines various passive and active targeting approaches, or multiple doses of various Tat-LNP formulations, may be advantageous in ensuring widespread HIV reactivation.

### Reservoir diversity and its impact on Tat efficacy

The HIV reservoir is highly heterogeneous with the vast majority of proviruses defective, harboring large internal deletions, hypermutations or inversions (126, 127). Furthermore, not all intact proviruses appear to be capable of reactivating (127). HIV cure efforts must therefore be focused on eliminating intact and inducible proviruses as these are the source of viral rebound. Indeed, only 0.1-10 CD4+ T cells per million are estimated to harbor intact and inducible proviruses (128, 129). A further challenge is that infected cells can undergo clonal expansion through homeostatic proliferation or antigen stimulated proliferation, and different clones display diverse responsiveness to T-cell activation (130). Whether there is a need to target all intact proviruses to effectively reduce the rebound-competent reservoir remains unclear, however, recent data suggests that after many years of suppressive ART, there are fewer proviruses capable of reactivation (131).

At a minimum, an LRA must be able to sufficiently reactivate an infected cell that harbors intact proviruses and can be induced to reactivate, and therefore capable of recrudescence following cessation of ART. The ability of the T66-LNP to increase multiply-spliced HIV RNA to higher levels than PMA/ionomycin in CD4+ T cells from PWH on ART is highly encouraging (51). However, it will be important to also understand if activation of virus expression occurred in all infected cells or from a subset of infected cells. This could be addressed using the tat-rev induced limiting dilution assay (TILDA) or quantitative viral outgrowth assay (qVOA), to measure the frequency of cells that can be induced to express multiply-spliced HIV RNA or virions, respectively.

Although the HIV reservoir was typically thought as being transcriptionally silent, cell associated HIV RNA is almost always able to be detected in PWH on ART (17, 18, 132–135), and HIV protein can be occasionally detected (136, 137). As Tat primarily functions by binding to TAR, we hypothesize that this new LRA will be effective in cells that are already transcriptionally active, thereby expressing the TAR stem loop. Consistent with this hypothesis, T66-LNP *ex vivo* led to no increase in TAR expression, yet potent increase in multiply-spliced HIV RNA (51, 90). More recent studies have shown that only a minority of HIV DNA+ cells express cell associated HIV RNA and this reduces over time on ART with an increased number of proviruses detected in non-genic regions (131, 138, 139). This suggests that Tat as an LRA might be best combined with another LRA that can efficiently initiate HIV transcription, as demonstrated recently using a combination of conventional LRAs and T66-LNP (90).

A further challenge in using latency reversal to reduce the reservoir is that latently infected cells express pro-survival proteins and are resistant to cytotoxic T-cell killing (140–142). Indeed, no studies exploring the efficacy of Tat as an LRA to date have yet investigated whether potent latency reversal by Tat can reduce the size of the HIV reservoir. Studies exploring the use of proapoptotic drugs have shown promise in their ability to selectively kill HIV DNA+ cells (143, 144). Therefore, combining a Tat LRA with a pro-apoptotic drug may also be needed.

## Summary and conclusion

The HIV Tat protein is a promising novel agent for the reversal of HIV latency *ex vivo*. Despite clear advantages over classical LRAs, including a demonstrated ability to potently upregulate multiply-spliced HIV RNA and protein production, and an HIV-specific mechanism of action, several challenges remain in the translation of HIV Tat into a clinically applicable LRA. The use of Tat variants that alter the domains mediating cellular secretion and uptake show potential to avoid inflammation and widespread toxicities in various tissues. Whilst the use of nanoparticles has greatly reduced the concentration of extracellular Tat, methods of efficiently targeting these nanocarriers to CD4+ T cells and diverse tissues which harbour infected cells will need to be developed. Finally, future studies will need to determine whether Tat can reactivate the transcriptionally silent reservoir and whether this is necessary to induce viral remission off-ART.
